# Enamine/Transition Metal Combined Catalysis: Catalytic Transformations Involving Organometallic Electrophilic Intermediates

**DOI:** 10.1007/s41061-019-0267-y

**Published:** 2019-11-16

**Authors:** Samson Afewerki, Armando Córdova

**Affiliations:** Department of Natural Sciences, Mid Sweden University, 851 70 Sundsvall, Sweden

**Keywords:** Combined catalysis, Enamine catalysis, Transition metal catalysis, Amino catalysis, Organocatalysis

## Abstract

The concept of merging enamine activation catalysis with transition metal catalysis is an important strategy, which allows for selective chemical transformations not accessible without this combination. The amine catalyst activates the carbonyl compounds through the formation of a reactive nucleophilic enamine intermediate and, in parallel, the transition metal activates a wide range of functionalities such as allylic substrates through the formation of reactive electrophilic π-allyl-metal complex. Since the first report of this strategy in 2006, considerable effort has been devoted to the successful advancement of this technology. In this chapter, these findings are highlighted and discussed.

## Introduction

**Scheme 1:**
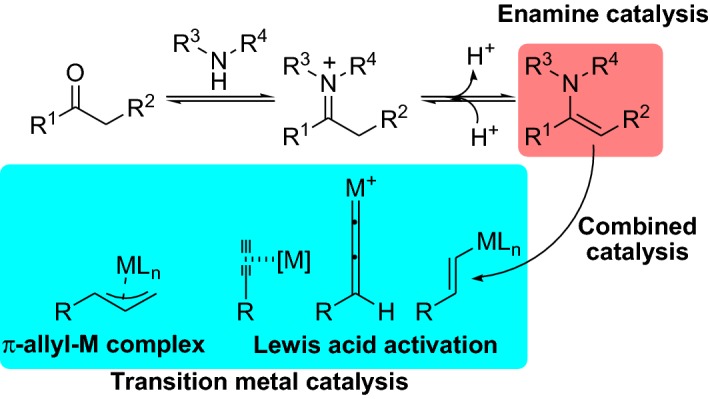
Illustration of combined enamine and transition metal catalysis, where the carbonyl compound and the amine provide the enamine intermediate and the transition metal can activate a wide range of substrates simultaneously, providing reactive electrophilic intermediates such as π-allyl-M complex, or electrophilic activation of an alkyne moiety or an alkenyl

The use of a small organic molecule to transform a chemical reaction through a catalytic approach (organocatalysis) has proved to be a very fruitful and widely employed chemical strategy [1, 2]. In this context, the use of an amine catalyst for the activation of ketones and aldehydes by the formation of enamine [3] or iminium [4] intermediates allows for a wide range of chemical transformations to proceed. These strategies become even more interesting and further broadened when the amine catalyst is combined with a transition metal catalyst, allowing unprecedented chemical reactions to ensue [5]. In the case when the amine catalyst provides a nucleophilic enamine, the intermediate can react directly with an electrophilic component activated by the transition metal catalyst [6, 7]. Specifically, the amine-catalyzed enamine formation of carbonyl compounds proceeds through the condensation reaction between the carbonyl component and the amine catalyst providing a nucleophilic enamine intermediate (enamine catalysis). In parallel, the transition metal can activate a wide range of substrates through various activation modes (e.g., through the formation of a π-allyl-metal complex, through a Tsuji-Trost type allylic activation [8]) providing an electrophilic intermediate (transition metal catalysis). By combining the catalytic cycles of these two intermediates (combined catalysis), a wide range of novel reactions can proceed (Scheme 1). For instance, the employment of gold catalysis for the electrophilic π-activation of alkynes has proven compatible with nucleophilic enamine addition to the triple bond [9]. Generally, amine catalysts are stable in most reaction conditions; however, when they are merged with metal catalysts, special caution need to be taken when considering the metal used, for instance, the use of copper might needed under inert atmosphere [10].

This chapter will discuss chemical transformation involving enamine and metal catalysis. Moreover, reactions involving enamine catalysis in domino-, cascade, sequential fashion or through iminium enamine activation, for instance in dynamic kinetic asymmetric transformation, will also be highlighted [5–7, 11]. More specifically, chemical transformations such as direct α-allylic alkylations and α-alkyl alkylations of carbonyl compounds, reactions employing alkynes or non-activated olefins as substrates, reactions involving an oxidation step or the preparation of various carbocyclic compounds through combined catalysis will also be discussed. These catalytic reactions have generally several advantages, such as avoiding the use of preformed activated carbonyl nucleophiles and dry solvents. Thus, the reactions can be performed in laboratories that do not have the special equipment necessary for some types of organometallic chemistry (e.g., glove box) and are less moisture sensitive. In fact, sometimes a small amount of water (e.g., 10 mol %) and oxygen are necessary for the transformations to occur [10, 81].

## α-Allylic Alkylation of Aldehydes and Ketones

The first demonstration of a successful combination of catalytic enamine activation and transition metal catalysis was disclosed by Córdova et al. [12]. This concept has been shown to be a very powerful tool for a variety of chemical reactions [5]. With respect to the direct α-allylic alkylation (AAA) of aldehydes and ketones, the research group managed to obtain the corresponding alkylated product in moderate-to-high yields.

**Scheme 2:**
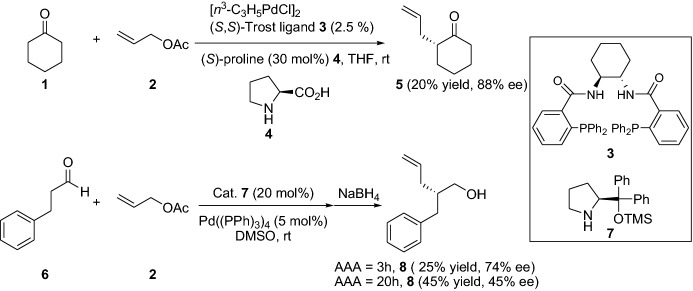
Selected examples from the first efforts of enantioselective α-allylic alkylation (AAA) by combined enamine and palladium catalysis for the generation of chiral ketone **5** and alcohol **8**

When investigating the enantioselective version, which was catalyzed by a chiral amine catalyst (e.g., **4** and **7**) in combination with an achiral or chiral ligand on the metal catalyst, the corresponding α-allylated products **5** and **8** were obtained in low yields with enantiomeric excesses (ee) of up to 88%. However, prolonged reaction times led to a decrease in the ee of the aldehyde-derived products (Scheme 2). These first examples of chiral amine/Pd-co-catalyzed enantioselective transformations were disclosed in this work but the reviewer requested that they not be specifically pointed out. Thus, they were put in the supporting information. However, the group was able to develop a reaction that gave the α-allylic alkylated aldehydes in high yields and ees using the same catalyst system [14]. It is noteworthy that, prior to this seminal work, it was often believed that the Lewis acid (metal catalyst) would most likely deactivate or inhibit the amine catalyst, and therefore it would be hard to accomplish a cooperative catalyst system [13]. Moreover, new avenues within the field of merging enamine and metal catalysis were opened.

**Scheme 3:**
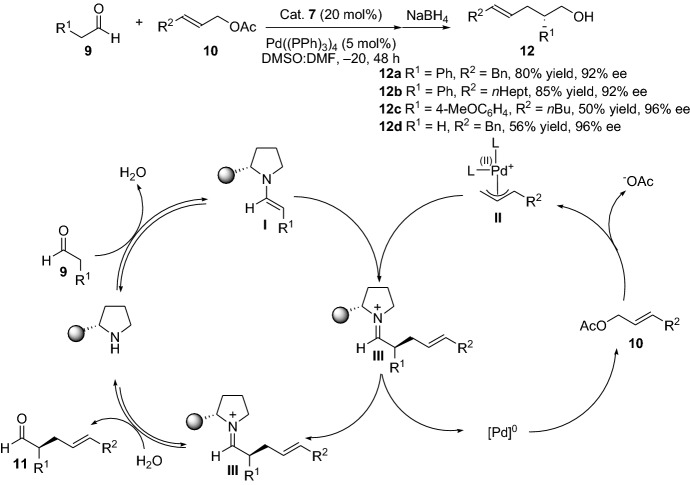
Highly enantioselective α-allylic alkylation (AAA) by combined enamine and palladium catalysis, and the proposed reaction mechanism

As mentioned above, the group later demonstrated a solid protocol for the generation of highly enantioselective alkylated products **12** by optimizing the previous protocol (Scheme 3) [14]. Interestingly, after a wide range of optimization with respect to parameters such as solvent, temperature and reaction time, the group found that having the right solvent system and temperature were critical in order to obtain high reactivity and simultaneously high enantioselectivity. Consequently, the optimal reaction conditions for the enantioselective transformation turned out to be a 1:1 mixture of DMSO (providing the highest reactivity) and DMF (providing highest enantioselectivity) at −20 °C for 48 h. The group also highlighted the importance of degassing the solvent with nitrogen gas prior to use for a successful reaction to occur, probably due to interference from the oxygen present in the solvent. The authors suggest a plausible mechanism for the chemical transformation, as depicted in Scheme 3. The transformation proceeds by a condensation step between the aldehyde **9** and amine catalyst **7**, providing the chiral enamine **I** intermediate, which undergoes a nucleophilic addition to the parallel generated electrophilic allylic intermediate **II**, generating the chiral coupled intermediate **III**. After subsequent hydrolysis, the chiral amine catalyst **7** is regenerated, and the chiral aldehyde **11** is obtained (Scheme 3). In 2007, List and colleagues [15] disclosed a direct α-allylic alkylation reaction with α-branched aldehydes as substrates. One of the key components was the employment of a chiral phosphoric acid as the cocatalyst in combination with an achiral amine catalyst. The authors termed the strategy as asymmetric counteranion-directed catalysis (ACDC) based on their proposed mechanism of action, where the electrophilic palladium species is charged, and a chiral counter anion surrounding the species promotes the selective attack of an achiral enamine.

**Scheme 4:**
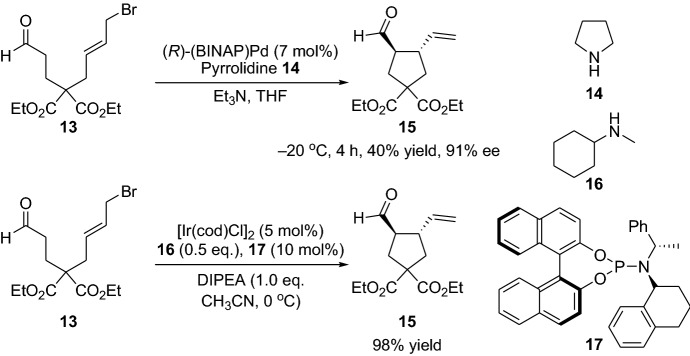
Selected examples from the integrated enamine and metal catalysts for the preparation of five- and six membered cyclization products, where the metal could be either palladium or iridium catalyst

**Scheme 5:**
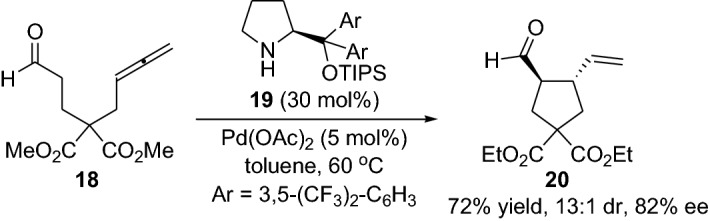
Example of the stereoselective intramolecular α-allylic alkylation using aldehyde-linked allenes providing carbocycle **20**

Furthermore, Saicic et al. employed the strategy of merging the enamine and metal catalysis for the preparation of five- and six membered rings by using either palladium [16] or iridium [17] catalysts (Scheme 4). The applicability of the intramolecular α-allylic alkylation stratagem was demonstrated for the stereoselective synthesis of the natural product (+)-allokainic acid [18]. Afterwards, Dixon et al. [19] disclosed the stereoselective intramolecular α-allylic alkylation using aldehyde and ketone-linked allenes. After a thorough optimization study based on several parameters such as amine and palladium catalysts, solvent and reaction time, the carbocyclic product **20** could be afforded in high yields and diastereoselectivities, and with up to 82% ee (Scheme 5). Later, several other groups employed allene-based substrates successfully in combination with gold- and enamine catalysis [20, 21]. All these reactions opened up new avenues and sparked great interest in the field, leading to the invention of a plethora of various α-allylic alkylation transformations employing ketones and aldehydes with a wide range of functionalities, substrates, catalysts and different conditions, providing the chemical community with a library of important tools [22–28].

**Scheme 6:**
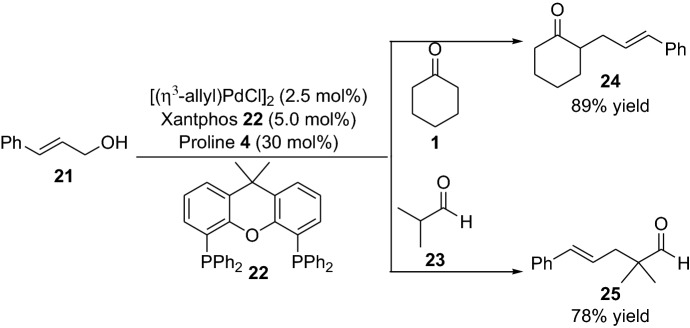
Selected examples from the first report of the α-allylic alkylation of ketone **1** and aldehyde **23** with the allylic alcohol **21**

**Scheme 7:**
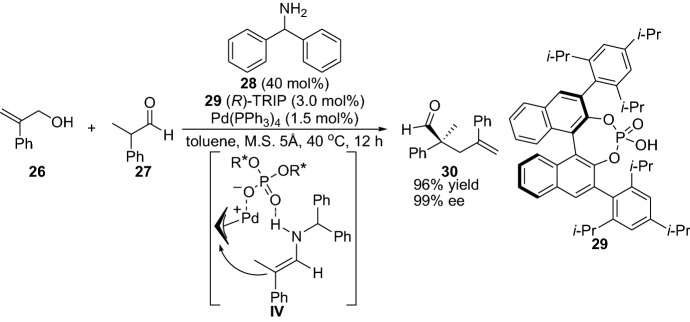
Stereoselective α-allylic alkylation of branched aldehyde **27** and allylic alcohol **26**

Another important milestone within the subject is the demonstration of the employment of allylic alcohols as substrates for the reaction with aldehydes and ketones established by Breit et al. [29]. Interestingly, although a chiral catalyst was tried during the screening studies, only racemic allylic products were obtained. However, the devised protocol provided allylic products in high yields (Scheme 6). This influential work demonstrated that simple allylic alcohols could be activated, thus avoiding the conversion to a better leaving group such as the transformation of the alcohol to an acetate or halide. Later, List et al. [15] employed the more challenging branched aldehydes for similar chemical transformation providing the corresponding product in high yield and with excellent enantioselectivity. The catalytic reaction was proposed to proceed via the formation of the combined enamine and allylic intermediate **IV** (Scheme 7) [30].

The employment of allylic alcohols in the α-allylic alkylation by combined catalysis (enamine and transition metal catalysis) has also been expanded by several other groups [31]. For example, Bandini et al. [32] used gold as the co-catalyst, Yasuda et al. [33] and Yoshida et al. [34, 35] used palladium as co-catalyst, and Zhou et al. [36] employed β-ketocarbonyl compounds as substrates.

**Scheme 8:**
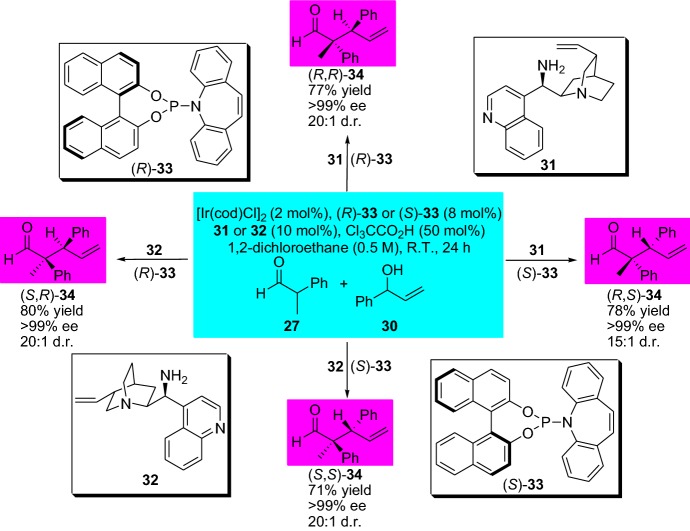
Combined enamine and transition metal catalysis for highly efficient and selective stereodivergent synthesis

The strategy of combined catalysis serves as a powerful tool allowing the stereodivergent synthesis (diastereo- and enantiodivergent catalyzed reactions) of various valuable compounds with multiple stereocenters, and further expands and diversify the chemical space [37, 38]. In this context, Carreira’s group disclosed an elegant strategy for the stereodivergent preparation of α-allylated aldehydes **34** by the concurrent combined activation of the nucleophile and electrophile using distinct catalysts. Remarkably, by simple alteration of the employed catalyst combinations, various aldehydes **34** could be generated with excellent stereoselectivities and efficiency (Scheme 8) [39]. The allylic alcohols **30** were activated by the chiral iridium complex catalyst (Ir/olefin), which was combined with a Brønsted acid promoter, and the branched aldehydes **27** by the chiral cinchona-alkaloid-derived primary amine catalysts **31** and **32**, respectively, forming chiral enamine intermediates. Interestingly, by following a similar stereodivergent strategy, and changing the chiral amine catalyst to a silyl protected secondary diarylprolinol and using dimethylhydrogen phosphate as the promoter, the Carreira group was able to prepare α-allylated linear aldehydes with high ee [40]. As a proof of concept, the strategy was employed for the enantioselective preparation of the antidepressant (−)-paroxetine. In the same year, the steroedivergent total synthesis of Δ^9^-tetrahydrocannabinols was also disclosed [41]. The preeminence of the disclosed strategy could also be employed for the stereodivergent α-allylation of protected α-amino and α-hydroxyacetaldehydes, providing important structural products for further use [42].

**Scheme 9:**
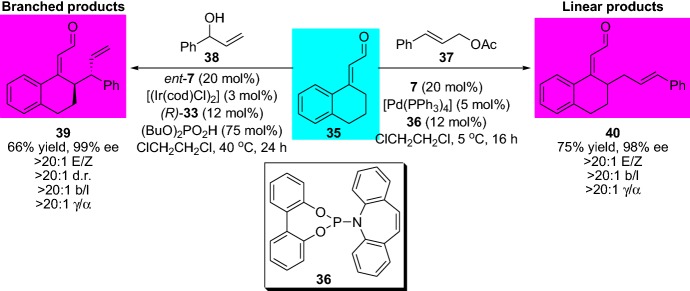
Combined enamine and transition metal catalysis for the highly efficient and selective diastereodivergent asymmetric γ-allylation

Later, Jørgensen et al. expanded the strategy by employing α,β-unsaturated aldehydes [43]. In this latter report, the authors developed a protocol for the preparation of both linear **40** and branched **39** products, which could be controlled by the type of transition metals and allylic substrates employed. The linear product could be generated by the use of allyl acetate **37** as the starting material and palladium as the metal catalyst, whilst, the branched product was achieved by using allylic alcohol **38** and iridium catalyst, respectively. The products were generated with good yields and excellent regio- and stereoselectivity (Scheme 9) [43]. Moreover, in 2011, Alexakis et al. [44] employed allylic alcohols by a one-pot procedure in the combined iridium and chiral amine catalysts. The chemical strategy proceeds by sequential iridium-catalyzed isomerization and subsequently stereoselective enamine addition providing acyclic α,β-chiral aldehydes.

## Combined Enamine and Metal Catalysis Using Alkynes as Substrates

**Scheme 10:**
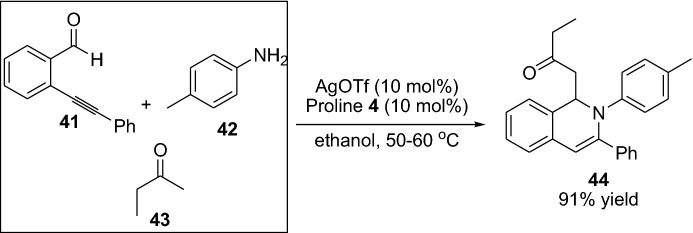
Multicomponent reaction providing the cyclic product **44**

**Scheme 11:**
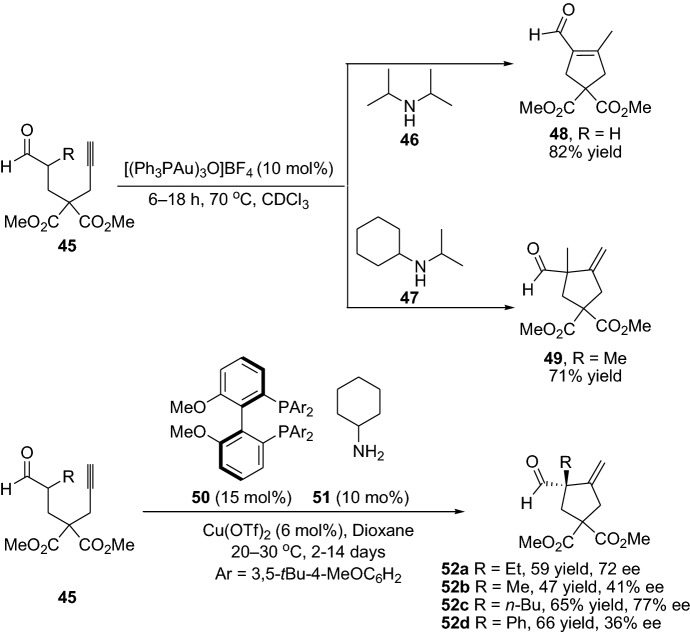
Selected examples from the direct α-functionalization of aldehydes and alkynes, and the enantioselective version of the chemical transformation

In the context of employing alkynes as substrate, in 2007, Ding and Wu [45] reported the employment of alkynes integrated with silver and enamine combined catalysis for the preparation of cyclic product **44** through a multicomponent reaction (Scheme 10). Shortly after, Kirsch et al. [46] disclosed the direct carbocyclization of aldehydes with alkynes by a combined gold and amine catalysts system. Interestingly, by altering the amine catalyst employed, the generation of the final product could be controlled, providing either product **48** or **49**. Product **48** proceeds through 5-exo-dig cyclization transformation and the product **49** through cyclization, followed by a double-bond migration step (Scheme 11). Furthermore, within this topic, Ratovelomanana-Vidal, Michelet and coauthors have been very fruitful and developed several chemical transformation employing alkynes as substrates, and various metals integrated with enamine catalysis providing various carbocyclic products. Here, they successfully employed Indium catalyst [47–49], copper catalyst [50–52], and iron catalyst [53]. However, it was not until 2012 that they demonstrated an enantioselective version of the chemical transformation, providing the chiral cyclopentanes **52** in moderate-to-high yields and ee (Scheme 11) [52].

**Scheme 12:**
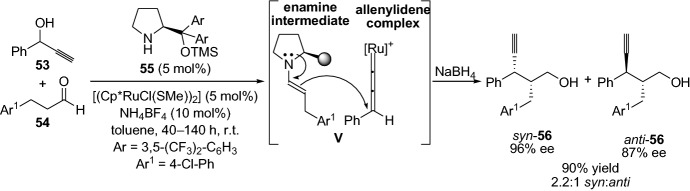
Enantioselective propargylic alkylation of propargylic alcohol and aldehydes

Moreover, the group of Nishibayashi has extensively employed propargylic alcohols [54–56] and esters [57] together with aldehydes and a combination of amine catalyst and the transition metal ruthenium or copper for the propargylic alkylation and allylation reactions. For instance, in 2010, they demonstrated the well-designed enantioselective propargylic alkylation with propargyl alcohol **53** and aldehydes **54**. Fascinatingly, the reaction proceeds via enamine nucleophilic addition to the ruthenium-allenylidene complex (**V**), providing the propargylic alkylated products **56** with high yields and enantioselectivity, and with the two diastereomers (*syn*-**56** and *anti*-**56**) (Scheme 12) [54].

**Scheme 13:**
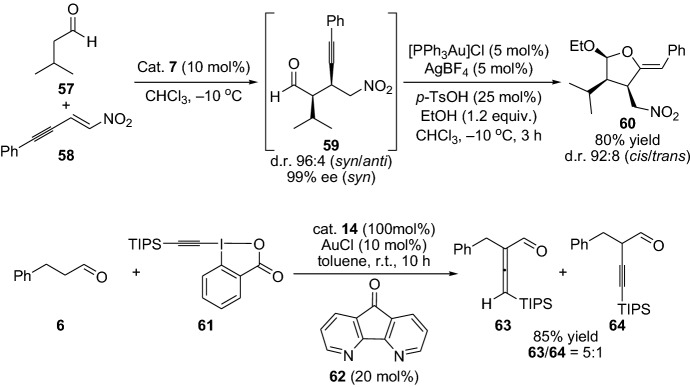
Enantioselective one-pot gold and enamine catalysis acetylation/cyclization reaction and the direct α-vinylidenation of aldehyde **6**

**Scheme 14:**
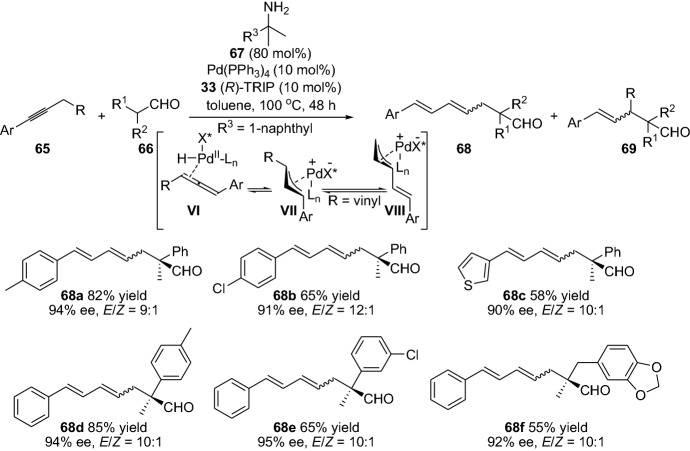
Asymmetric α-allylation of aldehydes with alkynes by merging chiral hydridopalladium and enamine catalysis

Gold has proven valuable as a transition metal catalyst in activating alkyne moieties. In this context, Alexakis et al. [58] and Wang et al. [59] have fruitfully combined gold and enamine catalysis for the reactions of alkynes with aldehydes. The first report demonstrates the enantioselective acetalization/cyclization transformation. The one-pot reaction between isovaleraldehyde **57** and nitroenyne **58** in the presence of ethanol, a catalytic amount of chiral amine **7**, and gold catalysts provided tetrahydrofuranyl ether **60** in high yield and diastereoselectivity (Scheme 13). Interestingly, the one-pot approach provided higher yield compared to the sequential approach [58]. In the report from Huang et al. for the direct α-vinylidenation between the aldehyde **6** and alkyne compound **61**, provided a mixture of the α-allenyl aldehyde **63** and α-alkynylated aldehyde **65**. The reaction generally favored the α-allenylated product; however, the reaction provided the product with high yields (up to 88%) and worked smoothly for a wide range of aldehydes (Scheme 13) [59]. In 2015, Dong and colleagues devised a protocol for the catalytic α-alkenylation of ketone with internal alkynes by the employment of bifunctional ligand-assisted approach combined with rhodium catalysis [60]. A thorough optimization of the reaction conditions allowed the authors to control the selective generation of the α,β- or β,γ-unsaturated ketones. Moreover, recently, Gong’s group demonstrated an asymmetric α-allylation approach of aldehyde with alkynes by combining hydridopalladium and enamine catalysis [61]. The catalytic system comprised of an achiral palladium complex, primary amine **67** and a chiral phosphoric acid **33**. The reaction tolerated a wide range of alkynes **65** and aldehydes **66**, with various functionalities. The chemical transformation proceeds through the electrophilic π-allylpalladium intermediates (**VII** and **VIII**) combined with nucleophilic enamine intermediate. The reaction provided the chiral α-quaternary aldehydes **68** in high yields and enantioselectivity (Scheme 14). Furthermore, ynals have also been coupled with aldehydes for the preparation of stereoselective propargylic alcohols through a cross-aldol reaction employing combined enamine and copper catalysis [62].

## Reactions with Non-activated Olefins

**Scheme 15:**
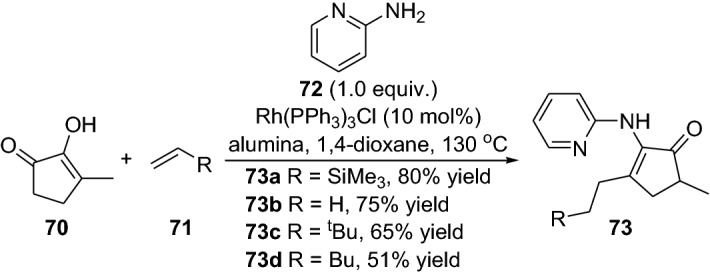
Intermolecular C-alkylation of 1,2-diketones **70** with simple olefins **71** employing an amine group as a recyclable directing group

**Scheme 16:**
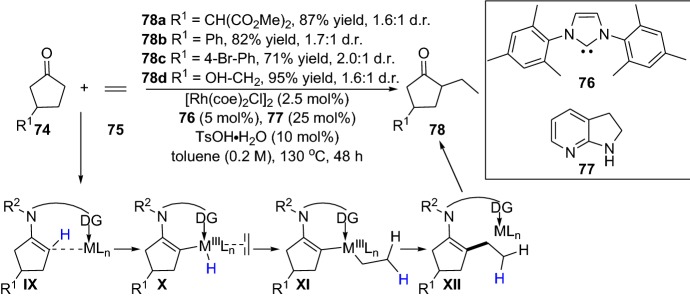
Regioselective α-alkylation of ketones **74** with ethylene **75** through combined rhodium and amine catalysts

**Scheme 17:**
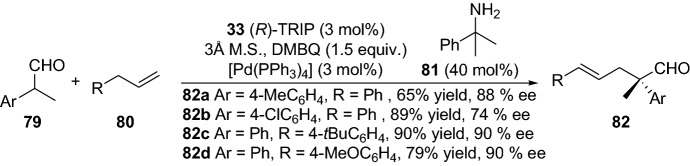
Enantioselective α-allylation of aldehydes **79** and terminal alkenes **80**

**Scheme 18:**
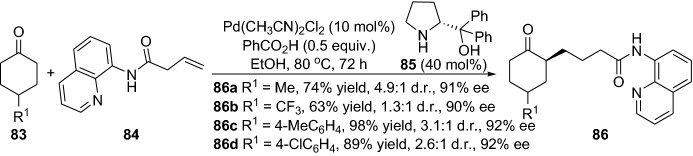
Enantioselective addition of cyclic ketones **83** to unactivated alkenes **84** generating the γ-addition products **86**

The reaction with simple non-activated olefins without pre-activation is a real challenge due to their inert nature making them resistant to most chemical reactions [63]. Within this theme, Dong and colleagues disclosed the intermolecular C-alkylation of 1,2-diketones **70** with simple olefins **71** by employing the recyclable and directing group aminopyridine **72** [64]. The aminopyridine reacts with the ketone, forming an enamine intermediate, and the rhodium catalyst promotes C–H vinyl bond activation. Noteably, the reaction tolerated a wide range of simple olefins with various ketones, providing alkylated products **73** in moderate-to-high yields (Scheme 15). It is noteworthy that the directing group can be cleaved and recycled; moreover, the reaction could be performed in one pot [64]. The same group further expanded their strategy, devising an elegant protocol for the regioselective α-alkylation of ketones with olefins [65]. The chemical reaction ensued by oxidative addition of the enamine and the C–H bond (**IX**), migratory insertion (**X**), migratory insertion into the olefin (**XI**), reductive elimination of the C–C bond (**XII**) and then further enamine hydrolysis provided the alkylated products **78** (Scheme 16). By using the simple ethylene **75** together with various functionalized ketones **74**, the products **78** were afforded in moderate-to-high yield (Scheme 16). The practical protocol was scalable and also worked well with other simple olefins [65]. Interestingly, very recently, the same group demonstrated an intermolecular direct branched-selective α-alkylation, providing β-branched ketones with excellent branched selectivity in an atom- and step economic approach [66]. Kang and colleagues also demonstrated the synergistic employment of combined rhodium and simple chiral amine catalysts in the enantioselective Michael addition of cyclic ketones with α,β-unsaturated 2-acyl imidazoles [67]. Furthermore, Gong’s group devised a chemical reaction in the first enantioselective α-allylation of aldehydes with terminal alkenes using asymmetric counter-anion catalysis and palladium-catalyzed allylic C–H activation combined with enamine catalysis [68]. The coupling between α-branched aromatic aldehydes **79** and terminal alkenes **80** delivered the chiral allylated products **82** in high yield, and with moderate-to-high enantioselectivity (Scheme 17). Additionally, very recently, the same group employed a combination of palladium catalyst integrated with the chiral amine **85** to prepare γ-coupled product **86** (Scheme 18) [69]. In 2014, Lei and colleagues reported a strategy for the C–H and C–H oxidative coupling of allylarenes with unactivated ketones by combining palladium and an enamine catalytic approach in the presence of the oxidant *p*-benzoquinone [70].

## Transition Metal- and Amine-Catalyzed Carbocyclization Reactions

**Scheme 19:**
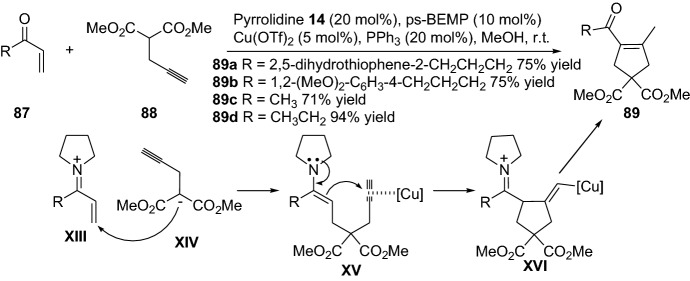
Combined iminium, enamine and copper cascade catalysis

**Scheme 20:**
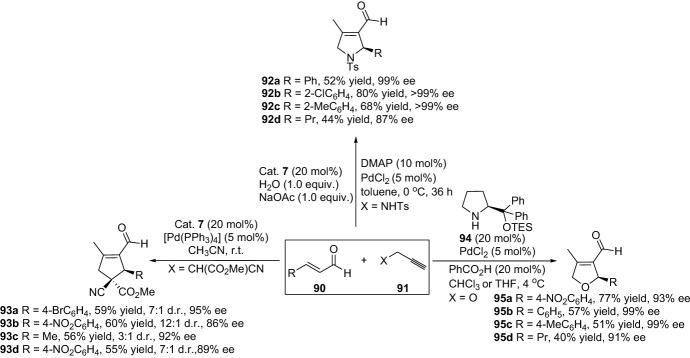
Preparation of various multifunctionalized carbocyclopentenes

**Scheme 21:**
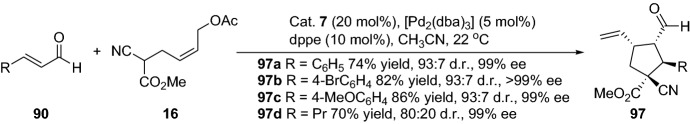
Enantioselective dynamic cascade reaction for the preparation of polysubstituted carbocycles including quaternary carbon stereocenter

Ensuing the vide supra highlighted work by the groups of Saicic et al. and Kirsch et al., combining enamine and transition metal catalysis for the generation of carbocyclic products through intramolecular transformation. The strategy has been further expanded through an intermolecular version. In this context, Dixon and coworkers demonstrated a chemical transformation for the production of carbocycles [71]. The cascade process proceeds through the formation of iminium intermediate **XIII**, which undergoes Michael addition with **XIV**, forming enamine and a transition metal activated intermediate **XV**, and, lastly, after protonolysis and hydrolysis of intermediate **XVI**, product **89** is afforded (Scheme 19). The chemical transformation generated the corresponding cyclopentenes **89** in moderate-to-high yield. Furthermore, several other groups have disclosed various chemical strategies with various substrates for the generation of these types of carbocyclopentenes in multisubstitution fashion. For instance, Wang’s group demonstrated a chemical reaction for the preparation of 2,5-dihydropyrroles **92** [72], and that of Córdova for the preparation of cyclopentenes **93** [73] and dihydrofurans **95** through a dynamic kinetic asymmetric transformation (DYKAT) approach (Scheme 20) [74]. The strategy was further extended by Córdova and colleagues through the use of heterogeneous transition metal catalysts [75–80] or the integration of an oxidation step [76]. The group further investigated the mechanism of palladium and amine co-catalyzed carbocyclization reaction through combined density functional theory (DFT) calculations and experiments [81]. In 2013, Córdova and colleagues devised a highly efficient protocol for the preparation of polysubstituted carbocycles with a quaternary carbon stereocenter [82]. The carbocyclic products **97** were afforded with high yields and diastereoselectivity and excellent enantioselectivity (Scheme 21).

**Scheme 22:**
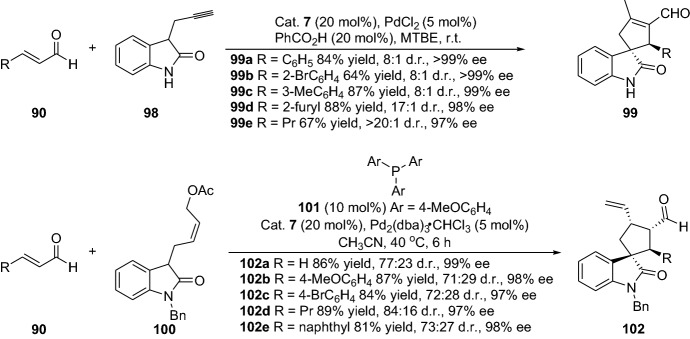
Stereoselective preparation of spirocyclic oxindoles through combined palladium and chiral amine catalysts

Wang and colleagues disclosed a chemical transformation for the preparation of spirocyclopentene oxindoles through combined palladium and chiral amine catalysis by employing alkyne-based substrates **198** [83]. In contrast, Córdova and colleagues employed allyl acetate-based substrates **100**, proceeding through a DYKAT process that generated polysubstituted spirocyclic oxindoles **102** [84]. Both the protocols presented provided structurally interesting compounds with high-to-excellent stereoselectivity and efficiency (Scheme 22). Furthermore, the group of Jørgensen employed a decarboxylative [4 + 2] cycloaddition strategy by merging palladium and amine catalysts for the preparation of vinyl tetrahydroquinolines [85]. Coupling between vinyl benzoxazinanones and α,β-unsaturated aldehydes, ensuing through iminium ion and enamine activation by the amine catalyst and simultaneous palladium-π-allyl complex activation of the vinyl benzoxazinanone, provided vinyl tetrahydroquinolines in good-to-high yields and with excellent stereoselectivity (up to 92% yield, > 98% ee and > 20:1 d.r.).

**Scheme 23:**
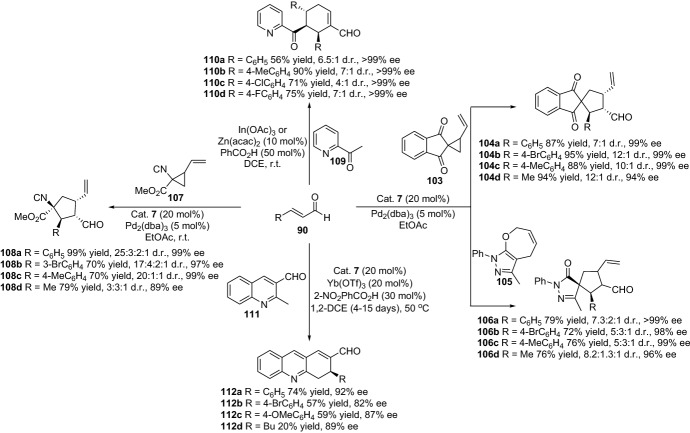
Various chemical strategies for the preparation of various carbocyclic compounds

Furthermore, the group of Rios also successfully disclosed various strategies for the preparation of carbocycles type molecules. For instance, through the enantioselective ring expansion of vinyl cyclopropanes, providing highly substituted spirocyclopentanes **104** [86], formal ring contraction for the generation of spiropyrazolones **106** [87], the asymmetric synthesis of cyclopentanes with four stereogenic centers **108** [88], the enantioselective acetyl aza-arene addition to α,β-unsaturated aldehydes affording chiral 2-acyl pyridines and pyrazines **110** [89], or, very recently disclosed, the highly enantioselective cascade reaction for the synthesis of dihydroacridines **112** (Scheme 23) [90].

## Miscellaneous Reactions

**Scheme 24:**
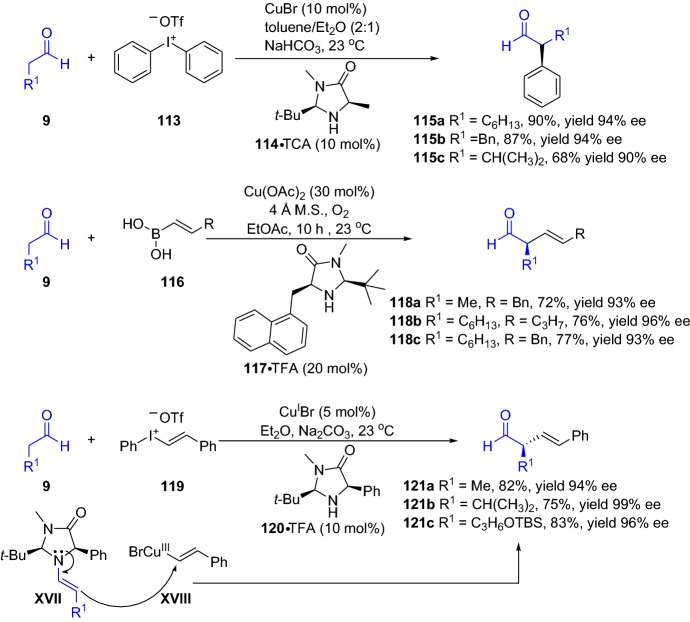
Enantioselective α-arylation, α-alkenylation and α-vinylation

The group of MacMillan have devised several protocols by integrating enamine and copper catalysis; for instance, the enantioselective α-arylation of aldehydes integrated with iodonium salts [91], enantioselective α-alkenylation of aldehydes with boronic acids [92], and in the enantioselective α-vinylation of aldehydes merged with vinyl iodonium triflate salts [93]. All the presented chemical transformations provided the coupled products **115**, **118** and **121** in high yield and high-to-excellent enantioselectivity (Scheme 24). The reaction cycle proceeds via coupling between the chiral enamine intermediate **XVII** and copper intermediate **XVIII**. Interestingly, during the catalytic cycle, the copper catalyst is altered between Cu(I)/Cu(III).

**Scheme 25:**
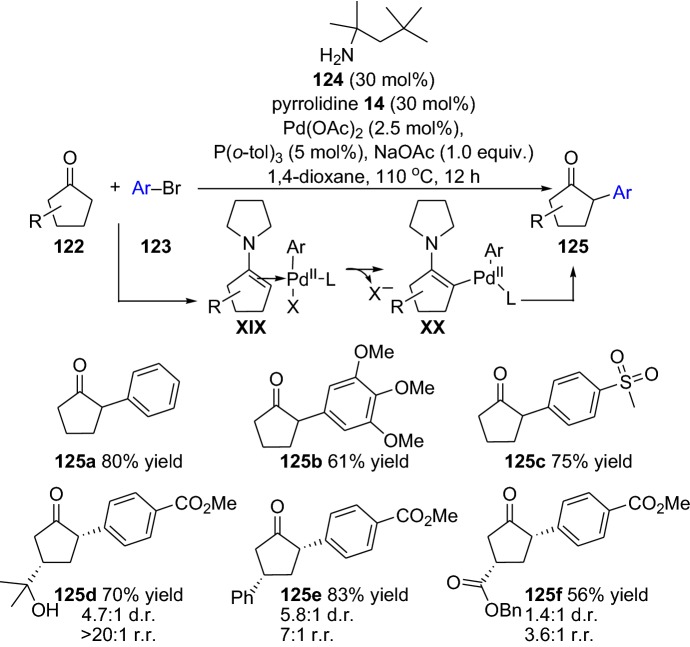
Selected examples from the direct α-arylation through combined enamine and palladium cooperative catalysis

Furthermore, the power of the enamine/transition metal combined catalysis have also made it possible to address the challenging α-arylation reactions of carbonyl compounds [94, 95]. Here, Dong’s group demonstrated the direct mono-α-C–H arylation of cyclopentanones **122** with aryl bromides **123** [96]. The devised chemical transformation overcome the challenges with the direct addition to the carbonyl moiety, self-aldol condensation, and with multiarylation promises. This was possible through the combined enamine and palladium cooperative catalysis proceeding through the intermediates **XIX** and **XX**, providing arylated products **125** with high selectivity and yield (Scheme 25). The chemical reaction tolerated a wide range of cyclopentanones and aryl moieties with various functionalities (Scheme 25). Moreover, the practicality of the devised protocol was also demonstrated by successfully scaling up the reaction to gram-scale, which provided the arylated product in high yield (72%) [96].

**Scheme 26:**
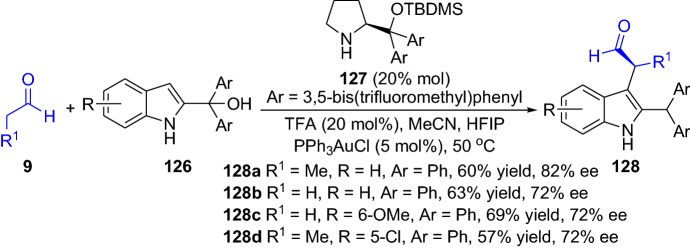
Asymmetric α-arylation of aldehydes and 2-Indolylmethanols

**Scheme 27:**
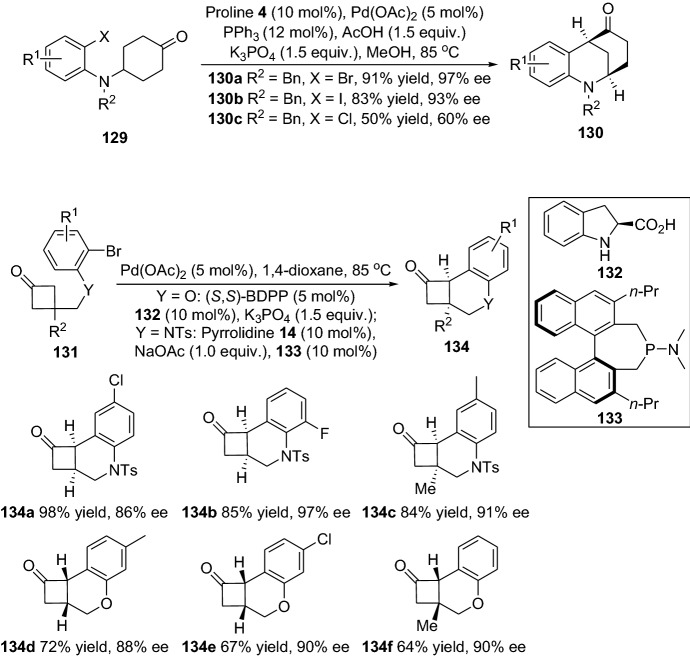
Combined palladium and amine catalyzed enantioselective α-arylative desymmetrization of cyclohexanones **129** and of cyclobutanones **131**

In 2018, Shi and colleagues devised an asymmetric version of the α-arylation reaction of aldehydes **9** employing 2-Indolylmethanols **126** as arylation agents (Scheme 26) [97]. Nevertheless, the transition metal used was gold, and the arylated products **128** were afforded in moderate-to-good yields and enantiomeric ratios (up to 69% yield and 82% ee) (Scheme 26). Furthermore, a desymmetrization strategy also employed cyclohexanones [98] and cyclobutanones [99] for the enantioselective synthesis of α-arylated products. In a report from Jia and colleagues, the combined catalyst system employed was palladium acetate (Pd(OA)_2_) with proline **4** as the amine catalyst, which provided optically active morphan derivatives containing α-carbonyl stereocenter **130**. The α-arylated compounds were afforded in high-to-excellent yields and enantioselectivities (up to 96% yield and 99% ee) (Scheme 27). Furthermore, the scale-up test of the chemical reaction provided the product **130** in 97% yield and with 98% ee [98]. This was further confirmed by Lu and collegueas, who reported that desymmetrization of the cyclobutanones **131** proceeded through an enantioselective intramolecular α-arylation, which provided the structurally interesting compounds **134** found in many bioactive natural products (Scheme 27). The reaction tolerated both O- and N-tethered aryl bromides, and an array of substrate scope was demonstrated successfully with a wide range of functionalities [99].

**Scheme 28:**
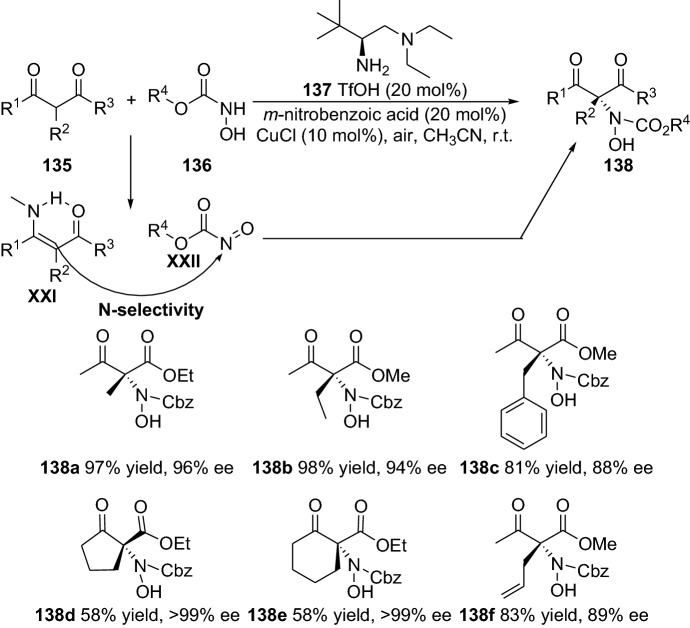
Combined transition metal and enamine catalysis integrated with aerobic oxidation

**Scheme 29:**
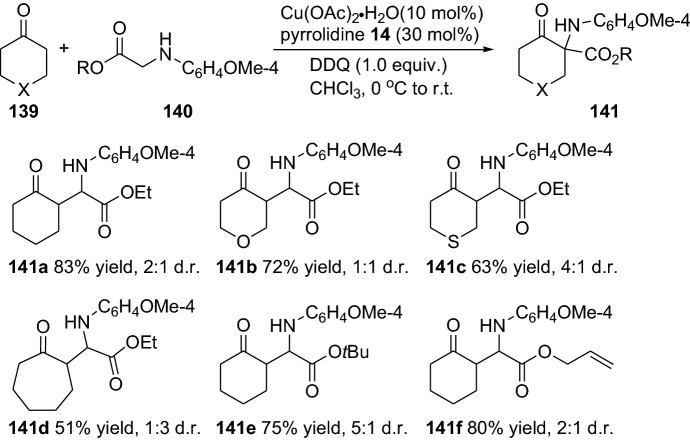
Combined transition metal and enamine catalysis in the cross-dehydrogenative coupling reaction

Additionally, the rare transition metal Niobium (Nb) as NbCl_5_ have fruitfully been merged with a primary amine’s enamine activation in the well-known Biginelli reaction [100]. Moreover, the stereoselective reaction presented by Xu and colleagues generates dihydropyrimidiones in moderate to good enantioselectivity (up to 84% ee) and with moderate-to-excellent efficiency (up to 99% yield) [101]. A further interesting strategy is the integration of oxidation steps in the enamine and transition metal combined catalysis [102]. In this regard, the group of Luo disclosed the merging of aerobic oxidation and enamine catalysis for the enantioselective synthesis of β-ketocarbonyls through an α-amination step [103]. The reaction showed a favored N-selectivity and ensue through the enamine **XXI** addition to the oxidized intermediate **XXII** (Scheme 28). The chemical transformation tolerated a wide range of functionalities and provided the α-aminated products **138** with moderate-to-high yields and enantioselectivities (Scheme 28). Continuing this subject, a similar strategy can be employed in the cross-dehydrogenative coupling reactions, through a combination of copper and pyrrolidine catalysts and an oxidant [104]. Although the reaction worked smoothly and provided the products **141** with moderate-to-high yields and with moderate diastereoselectivity, the asymmetric version provided very low enantioselectivity (up to 15%) (Scheme 29). The authors proposed a chemical transformation to proceed via a radical single electron transfer (SET) activating substrate **140** combined with enamine activation of the cyclic ketones **139** [104]. Moreover, several other reports have also demonstrated the enamine and transition metal combined catalysis involving an oxidation step [105], such as the report by Xu and colleagues in the catalytic enantioselective oxidative α-C–H and *N*,*O*-ketalization of ketones by merging primary amine and copper catalysts [106], oxidative coupling merging vanadium and enamine catalysis by Sud and colleagues [107]. Furthermore, several other groups have demonstrated the successful employment of a combined copper or iridium and enamine catalysis strategy in the highly stereoselective α-alkylation of aldehydes [108], in the enantioselective alkylation of cyclic *N*-acyl hemiaminals with aldehydes [109], in the multifunctionalization of unactivated cyclic ketones [110], and in the α-amination of aldehydes [111].

## Conclusion

To date, the chemical community has witnessed the fruitful growth of the powerful strategy of combining enamine and transition metal catalysis. Starting from the first examples in 2006, demonstrating the possibility of overcoming any inhibition or quenching by concomitantly merging simple amine catalysts, providing nucleophilic enamine coupled with transition metal activated electrophilic intermediate. Endeavors to further expand the strategy have allowed a plethora of novel chemical reactions with a wide range of simple starting materials to proceed in atom- and step-economic manner. Furthermore, we have seen novel strategies adopting the stereodivergent preparation of a wide range of important compounds with multiple stereocenters and with diversified functionalities, which additionally expands the chemical space. We believe this strategy will continue emerging into new innovative reactions allowing the coupling of more challenging components with indefinite chemical transformations.

## References

[1] Dalko PI, Moisan L (2001). Enantioselective organocatalysis. Angew Chem Int Ed.

[2] MacMillan DWC (2008). The advent and development of organocatalysis. Nature.

[3] Mukherjee S, Yang JW, Hoffmann S, List B (2007). Asymmetric enamine catalysis. Chem Rev.

[4] Erkkilä A, Majander I, Pihko PM (2007). Iminium catalysis. Chem Rev.

[5] Afewerki S, Córdova A (2016). Combinations of aminocatalysts and metal catalysts: a powerful cooperative approach in selective organic synthesis. Chem Rev.

[6] Wang H, Deng Y (2015) Cooperative enamine-lewis acid catalysis. In: Peters R (ed) Cooperative catalysis. Wiley-VCH, Weinheim, pp 111–144

[7] Afewerki Samson, Córdova Armando (2017). Cooperative Lewis Acids and Aminocatalysis. Chiral Lewis Acids in Organic Synthesis.

[8] Trost BM, Van Vranken DL (1996). Asymmetric transition metal-catalyzed allylic alkylations. Chem Rev.

[9] Lauterbach T, Asiri AM, Hashmi ASK, Pérez PJ (2014). Advances in organmetallic intermediates of gold catalysis. Advances in organometallic chemistry.

[10] Afewerki S, Breistein P, Pirttilä K, Deiana L, Dziedzic P, Ibrahem I, Córdova A (2012). Catalytic enantioselective β-alkylation of & #x03B1;, β-unsaturated aldehydes by combination of transition-metal- and aminocatalysis: total synthesis of bisabolane sesquiterpenes. Chem Eur J.

[11] Deng Y, Kumar S, Wang H (2014). Synergistic-cooperative combination of enamine catalysis with transition metal catalysis. Chem Commun.

[12] Ibrahem I, Córdova A (2006). Direct catalytic intermolecular α-allylic alkylation of aldehydes by combination of transition-metal and organocatalysis. Angew Chem Int Ed.

[13] Qiu R, Chen Y, Yin S-F, Xu X, Au C-T (2012). A mini-review on air-stable organometallic Lewis acids: synthesis, characterization, and catalytic application in organic synthesis. RSC Adv.

[14] Afewerki S, Ibrahem I, Rydfjord J, Breistein P, Córdova A (2012). Direct regiospecific and highly enantioselective intermolecular α-allylic alkylation of aldehydes by a combination of transition-metal and chiral amine catalysts. Chem Eur J.

[15] Mukherjee S, List B (2007). Chiral counteranions in asymmetric transition-metal catalysis: highly enantioselective Pd/Brønsted acid-catalyzed direct α-allylation of aldehydes. J Am Chem Soc.

[16] Bihelovic F, Matovic R, Vulovic B, Saicic RN (2007). Organocatalyzed cyclizations of π-allylpalladium complexes: a new method for the construction of five- and six membered rings. Org Lett.

[17] Vulovic B, Bihelovic F, Matovic R, Saicic RN (2009). Organocatalyzed Tsuji-Trost reaction: a new method for the closure of five- and six-membered rings. Tetrahedron.

[18] Vulovic B, Gruden-Pavlovic M, Matovic R, Saicic RN (2014). Substrate stereocontrol in the intramolecular organocatalyzed Tsuji-Trost reaction: enantioselective synthesis of allokainates. Org Lett.

[19] Li M, Datta S, Barber DM, Dixon DJ (2012). Dual amine and palladium catalysis in diastereo- and enantioselective allene carbocyclization reactions. Org Lett.

[20] Ballesteros A, Morán-Poladura P, González JM (2016). Gold(I) operational in synergistic catalysis for the intermolecular α-addition reaction of aldehydes across allenamides. Chem Commun.

[21] Fernández-Casado J, Nelson R, Mascareñas JL, López F (2016). Synergistic gold and enamine catalysis: intermolecular α-alkylation of aldehydes with allenamides. Chem Commun.

[22] Weix DJ, Hartwig JF (2007). Regioselective and enantioselective iridium-catalyzed allylation of enamines. J Am Chem Soc.

[23] Sato T, Tomioka K (2009). Catalytic asymmetric intramolecular allylation of aldehyde. Heterocycles.

[24] Zhao X, Liu D, Xie F, Liu Y, Zhang W (2011). Efficient palladium-catalyzed asymmetric allylic alkylation of ketones and aldehydes. Org Biomol Chem.

[25] Zhao X, Liu D, Guo H, Liu Y, Zhang W (2011). C–N bond cleavage of allylic amines via hydrogen bond activation with alcohol solvents in Pd-catalyzed allylic alkylation of carbonyl compounds. J Am Chem Soc.

[26] Cattopadhyay K, Recio A, Tunge JA (2012). Palladium-catalyzed, pyrrolidine mediated arylmethylation of ketones and aldehydes with coumarinyl(methyl) acetates. Org Biomol Chem.

[27] Yoshida M, Terumine T, Masaki E, Hara S (2013). Direct asymmetric α-allylation α branched aldehydes by two catalytic systems with an achiral Pd complex and a chiral primary α-amino acid. J Org Chem.

[28] Huo X, Quan M, Yang G, Zhao X, Liu D, Liu Y, Zhang W (2014). Hydrogen-bond activated palladium-catalyzed allylic alkylation via allylic alkyl ethers: challenging leaving groups. Org Lett.

[29] Usui I, Schmidt S, Breit B (2009). Dual palladium- and proline-catalyzed allylic alkylation of enolizable ketones and aldehydes with allylic alcohols. Org Lett.

[30] Jiang G, List B (2011). Direct asymmetric α-allylation of aldehydes with simple allylic alcohols enabled by the concerted action of three different catalysts. Angew Chem Int Ed.

[31] Huo X, Yang G, Liu D, Liu Y, Gridnev ID, Zhang W (2014). Palladium-catalyzed allylic alkylation of simple ketones with allylic alcohols and its mechanistic study. Angew Chem Int Ed.

[32] Chiarucci M, di Lillo M, Romaniello A, Cozzi PG, Cera G, Bandini M (2012). Gold meets enamine catalysis in the enantioselective α-allylic alkylation of aldehydes with alcohols. Chem Sci.

[33] Yasuda S, Kumagai N, Shibasaki M (2012). Direct asymmetric α-allylation of ketones with allylic alcohols via Pd/enamine cooperative function. Heterocycles.

[34] Yoshida M, Masaki E, Terumine T, Hara S (2014). Asymmetric α-allylation of α branched aldehydes with allyl alcohols by synergistic catalysis using an achiral palladium complex and a chiral primary amino acid. Synthesis.

[35] Yoshida M (2017). Asymmetric α-allylation of α-substituted β-ketoesters with allyl alcohols. J Org Chem.

[36] Zhou H, Zhang L, Xu C, Luo S (2015). Chiral primary amine/palladium dual catalysis for asymmetric allylic alkylation of β-ketocarbonyl compounds with allylic alcohols. Angew Chem Int Ed.

[37] Krautwald S, Carreira EM (2017). Stereodivergence in asymmetric catalysis. J Am Chem Soc.

[38] Beletskaya IP, Nájera C, Yus M (2018). Stereodivergent catalysis. Chem Rev.

[39] Krautwald S, Sarlah D, Schafroth MA, Carreira EM (2013). Enantio- and diastereodivergent dual catalysis: α-allylation of branched aldehydes. Science.

[40] Krautwald S, Schafroth MA, Sarlah D, Carreira EM (2014). Stereodivergent α-allylation of linear aldehydes with dual iridium and amine catalysis. J Am Chem Soc.

[41] Schafroth MA, Zuccarello G, Krautwald S, Sarlah D, Carreira EM (2014). Stereodivergent total synthesis of ∆^9^-tetrahydrocannabinols. Angew Chem Int Ed.

[42] Sandmeier T, Krautwald S, Zipfel HF, Carreira EM (2015). Stereodivergent dual catalytic α-allylation of protected α-amino- and α-hydroxyacetaldehydes. Angew Chem Int Ed.

[43] Næsborg L, Halskov KS, Tur F, Mønsted SMN, Jørgensen KA (2015). Asymmetric y allylation of α,β-unsaturated aldehydes by combined organocatalysis and transition-metal catalysis. Angew Chem Int Ed.

[44] Quintard A, Alexakis A, Mazet C (2011). Access to high levels of molecular complexity by one-pot iridium/enamine asymmetric catalysis. Angew Chem Int Ed.

[45] Ding Q, Wu J (2007). Lewis acid- and organocatalyst-cocatalyzed multicomponent reactions of 2-alkynylbenzaldehydes, amines, and ketones. Org Lett.

[46] Binder JT, Crone B, Haug TT, Menz H, Kirsch SF (2008). Direct carbocyclization of aldehydes with alkynes: combining gold catalysis with aminocatalysis. Org Lett.

[47] Montaignac B, Vitale MR, Michelet V, Ratovelomanana-Vidal V (2010). Combined InCl3- and amine-catalyzed intramolecular addition of α-disubstituted aldehydes onto unactivated alkynes. Org Lett.

[48] Montaignac B, Vitale MR, Ratovelomanana-Vidal V, Michelet V (2010). InCl3/CyNH2 cocatalyzed carbocyclization reaction: an entry to α-disubstituted exo-methylene cyclopentanes. J Org Chem.

[49] Praveen C, Montaignac B, Vitale MR, Ratovelomanana-Vidal V, Michelet V (2013). Enantioselective merger of aminocatalysis with π-Lewis acid metal catalysis: asymmetric preparation of carbo- and heterocycles. ChemCatChem.

[50] Montaignac B, Vitale MR, Ratovelomanana-Vidal V, Michelet V (2011). Cooperative copper(I) and primary amine catalyzed room-temperature carbocyclization of formyl alkynes. Eur J Org Chem.

[51] Montaignac B, Östlund V, Vitale MR, Ratovelomanana-Vidal V, Michelet V (2012). Copper(I)-amine metallo-organocatalyzed synthesis of carbo- and heterocyclic systems. Org Biomol Chem.

[52] Montaignac B, Praveen C, Vitale MR, Michelet V, Ratovelomanana-Vidal V (2012). Enantioselective metallo-organocatalyzed preparation of cyclopentanes bearing an all-carbon quaternat stereocenter. Chem Commun.

[53] Praveen C, Levêque S, Vitale MR, Michelet V, Ratovelomanana-Vidal V (2014). Synergistic iron-and-amine catalysis in carbocyclizations. Synthesis.

[54] Ikeda M, Miyake Y, Nishibayashi Y (2010). Cooperative catalytic reactions using organocatalysts and transition-metal catalysts: enantioselective propargylic alkylation of propargylic alcohols with aldehydes. Angew Chem Int Ed.

[55] Ikeda M, Miyake Y, Nishibayashi Y (2012). Cooperative catalytic reactions using organocatalysts and transition metal catalysts: propargylic allylation of propargylic alcohols with α, β-unsaturated aldehydes. Organometallics.

[56] Motoyama K, Ikeda M, Miyake Y, Nishibayashi Y (2011). Cooperative catalytic reactions using lewis acids and organocatalysts: enantioselective propargylic alkylation of propargylic alcohols bearing an internal alkyne with aldehydes. Eur J Org Chem.

[57] Yoshida A, Ikeda M, Hattori G, Miyake Y, Nishibayashi Y (2011). Cooperative catalytic reactions using organocatalysts and transition metal catalysts: enantioselective propargylic alkylation of propargylic esters with aldehydes. Org Lett.

[58] Belot S, Vogt KA, Besnard C, Krause N, Alexakis A (2009). Enantioselective one pot organocatalytic Michael addition/gold-catalyzed tandem acetalization/cyclization. Angew Chem Int Ed.

[59] Wang Z, Li X, Huang Y (2013). Direct α-vinylidenation of aldehydes and subsequent cascade: gold and amine catalysts work synergistically. Angew Chem Int Ed.

[60] Mo F, Lim HN, Dong G (2015). Bifunctional ligand-assisted catalytic ketone α-alkenylation with internal alkynes: controlled synthesis of enones and mechanistic studies. J Am Chem Soc.

[61] Su Y-L, Li L-L, Zhou X-L, Dai Z-Y, Wang P-S, Gong L-Z (2018). Asymmetric α-allylation of aldehydes with alkynes by integrating chiral hydridopalladium and enamine catalysis. Org Lett.

[62] Gómez-Bengoa E, García JM, Jiménez S, Lapuerta I, Mielgo A, Odriozola JM, Otazo I, Razkin J, Urruzuno I, Vera S, Oiarbide M, Palomo C (2013). Asymmetric synthesis of propargylic alcohols via aldol reaction of aldehydes with ynals promoted by prolinol ether-transition metal-Brønsted acid cooperative catalysis. Chem Sci.

[63] Dong Z, Ren Z, Thompson SJ, Xu Y, Dong G (2017). Transition-metal-catalyzed CH alkylation using alkenes. Chem Rev.

[64] Wang Z, Reinus BJ, Dong G (2012). Catalytic intermolecular *C*-alkylation of 1,2 diketones with simple olefins: a recyclable directing group strategy. J Am Chem Soc.

[65] Mo F, Dong G (2014). Regioselective ketone α-alkylation with simple olefins via dual activation. Science.

[66] Xing D, Qi X, Marchant D, Liu P, Dong G (2019). Branched-selective direct α-alkylation of cyclic ketones with simple alkenes. Angew Chem.

[67] Qurban S, Gong J, Du Y, Kang Q (2018). Rhodium(III)/amine synergistically catalyzed enantioselective Michael addition of cyclic ketones with α, β-unsaturated 2-acyl imidazoles. Org Chem Front.

[68] Wang PS, Lin HC, Zhai YJ, Han ZY, Gong LZ (2014). Chiral Counteranion strategy for asymmetric oxidative C(sp3)-H/C(sp3)-H coupling: enantioselective α-allylation of aldehydes with terminal alkenes. Angew Chem Int Ed.

[69] Shen H-C, Zhang L, Chen S-S, Feng J, Zhang B-W, Zhang Y, Zhang X, Wu Y-D, Gong L-Z (2019). Enantioselective addition of cyclic ketones to unactivated alkenes enabled by amine/Pd(II) cooperative catalysis. ACS Catal.

[70] Tang S, Wu X, Liao W, Liu K, Liu C, Luo S, Lei A (2014). Synergistic Pd/enamine catalysis: a strategy for the C–H/C–H oxidative coupling of allylarenes with unactivated ketones. Org Lett.

[71] Yang T, Ferrali A, Campbell L, Dixon DJ (2008). Combination iminium, enamine and copper(I) cascade catalysis: a carboannulation for the synthesis of cyclopentenes. Chem Commun.

[72] Sun W, Zhu G, Hong L, Wang R (2011). The marriage of organocatalysis with metal catalysis: access to multisubstituted chiral 2,5-dihydropyrroles by cascade iminium/enamine-metal cooperative catalysis. Chem Eur J.

[73] Zhao GL, Ullah F, Deiana L, Lin S, Zhang Q, Sun J, Ibrahem I, Dziedzic P, Córdova A (2010). Dynamic kinetic asymmetric transformation (DYKAT) by combined amine- and transition-metal-catalyzed enantioselective cycloisomerization. Chem Eur J.

[74] Lin S, Zhao GL, Deiana L, Sun J, Zhang Q, Leijonmarck H, Cordova A (2010). Dynamic kinetic asymmetric domino oxa-Michael/carbocyclization by combination of transition-metal and amine catalysis: catalytic enantioselective synthesis of dihydrofurans. Chem Eur J.

[75] Deiana L, Afewerki S, Palo-Nieto C, Verho O, Johnston EV, Córdova A (2012). Highly enantioselective cascade transformations by merging heterogeneous transition metal catalysis with asymmetric aminocatalysis. Sci Rep.

[76] Deiana L, Jiang Y, Palo-Nieto C, Afewerki S, Incerti-Pradillos CA, Verho O, Tai CW, Johnston EV, Córdova A (2014). Combined heterogeneous metal/chiral amine: multiple relay catalysis for versatile eco-friendly synthesis. Angew Chem Int Ed.

[77] Deiana L, Ghisu L, Córdova O, Afewerki S, Zhang R, Córdova A (2014). Efficient and highly enantioselective aerobic oxidation-Michael-carbocyclization cascade transformations by integrated Pd(0)-CPG nanoparticle/chiral amine relay catalysis. Synthesis.

[78] Xu C, Deiana L, Afewerki S, Incerti-Pradillos C, Córdova O, Guo P, Córdova A, Hedin N (2015). The use of porous palladium(II)-polyimine in cooperatively catalyzed highly enantioselective cascade transformations. Adv Synth Catal.

[79] Deiana L, Ghisu L, Afewerki S, Verho O, Johnston EV, Hedin N, Bacsik Z, Cordova A (2014). Enantioselective heterogeneous synergistic catalysis for asymmetric cascade transformations. Adv Synth Catal.

[80] Xu C, Afewerki S, Tai CW, Córdova A, Hedin N (2016). Cyclopalladated azo-linked porous polymers in C–C bond forming reactions. ChemistrySelect.

[81] Santoro S, Deiana L, Zhao G-L, Lin S, Himo F, Córdova A (2014). Mechanism of palladium/amine cocatalyzed carbocyclization of aldehydes with alkynes and its merging with “Pd Oxidase Catalysis”. ACS Catal.

[82] Ma G, Afewerki S, Deiana L, Palo-Nieto C, Liu L, Sun J, Ibrahem I, Córdova A (2013). A palladium/chiral amine co-catalyzed enantioselective dynamic cascade reaction: synthesis of polysubstituted carbocycles with a quaternary carbon stereocenter. Angew Chem Int Ed.

[83] Sun W, Zhu G, Wu C, Hong L, Wang R (2012). “Organo-Metal” synergistic catalysis: the 1 + 1>2 effect for the construction of spirocyclopentene oxindoles. Chem Eur J.

[84] Afewerki S, Ma G, Ibrahem I, Liu L, Sun J, Cordova A (2015). Highly enantioselective control of dynamic cascade transformations by dual catalysis: asymmetric synthesis of polysubstituted spirocyclic oxindoles. ACS Catal.

[85] Leth LA, Glaus F, Meazza M, Fu L, Thøgersen MK, Bitsch EA, Jørgensen KA (2016). Decarboxylative [4 + 2] cycloaddition by synergistic palladium and organocatalysis. Angew Chem Int Ed.

[86] Meazza M, Rios R (2016). Synergistic catalysis: enantioselective ring expansion of vinyl cyclopropanes combining four catalytic cycles for the synthesis of highly substituted spirocyclopentanes bearing up to four stereocenters. Chem Eur J.

[87] Meazza M, Kamlar M, Jašíková L, Formánek B, Mazzanti A, Roithová J, Veselý J, Rios R (2018). Synergistic formal ring contraction for the enantioselective synthesis of spiropyrazolones. Chem Sci.

[88] Zhang K, Meazza M, Izaga A, Contamine C, Gimeno MC, Herrera RP, Rios R (2017). Synergistic catalysis: asymmetric synthesis of cyclopentanes bearing four stereogenic centers. Synthesis.

[89] Meazza M, Sitinova G, Poderi C, Mancinelli M, Zhang K, Mazzanti A, Torres RR (2018). Synergistic catalysis: highly enantioselective acetyl aza-arene addition to enals. Chem Eur J.

[90] Meninno S, Meazza M, Yang JW, Tejero T, Merino-Gomez P, Rios R (2019). Synergistic catalysis: highly enantioselective cascade reaction for the synthesis of dihydroacridines. Chem Eur J.

[91] Allen AE, MacMillan DWC (2011). Enantioselective α-arylation of aldehydes via the productive merger of iodonium salts and organocatalysis. J Am Chem Soc.

[92] Stevens JM, MacMillan DWC (2013). Enantioselective α-alkenylation of aldehydes with boronic acids via the synergistic combination of copper(II) and amine catalysis. J Am Chem Soc.

[93] Skucas E, MacMillan DWC (2012). Enantioselective α-vinylation of aldehydes via the synergistic combination of copper and amine catalysis. J Am Chem Soc.

[94] Palucki M, Buchwald SL (1997). Palladium-catalyzed α-arylation of ketones. J Am Chem Soc.

[95] Hamann BC, Hartwig JF (1997). Palladium-catalyzed direct α-arylation of ketones. Rate acceleration by sterically hindered chelating ligands and reductive elimination from a transition metal enolate complex. J Am Chem Soc.

[96] Xu Y, Su T, Huang Z, Dong G (2016). Practical direct α-arylation of cyclopentanones by palladium/enamine cooperative catalysis. Angew Chem Int Ed.

[97] Xu M-M, Wang H-Q, Mao Y-J, Mei G-J, Wang S-L, Shi F (2018). Cooperative catalysis-enabled asymmetric α-arylation of aldehydes using 2-indolylmethanols as arylation reagents. J Org Chem.

[98] Liu R-R, Li B-L, Lu J, Shen C, Gao J-R, Jia Y-X (2016). Palladium/l-proline catalyzed enantioselective α-arylative desymmetrization of cyclohexanones. J Am Chem Soc.

[99] Wang M, Chen J, Chen Z, Zhong C, Lu P (2018). Enantioselective desymmetrization of cyclobutanones enabled by synergistic palladium/enamine catalysis. Angew Chem Int Ed.

[100] Nagarajaiah H, Mukhopadhyay A, Moorthy JN (2016). Biginelli reaction: an overview. Tetrahedron Lett.

[101] Cai Y-F, Yang H-M, Li L, Jiang K-Z, Lai G-Q, Jiang J-X (2010). Xu L-W (2010) Cooperative and enantioselective NbCl_5_/primary amine catalyzed biginelli reaction. Eur J Org Chem.

[102] Twilton J, Le C, Zhang P, Shaw MH, Evans RW, MacMillan DWC (2017). The merger of transition metal and photocatalysis. Nat Rev Chem.

[103] Xu C, Zhang L, Luo S (2014). Merging aerobic oxidation and enamine catalysis in the asymmetric & α-amination of β-ketocarbonyls using N-hydroxycarbamates as nitrogen source. Angew Chem Int Ed.

[104] Xie J, Huang Z-Z (2010). Cross-dehydrogenative coupling reactions by transition-metal and aminocatalysis for the synthesis of amino acid derivatives. Angew Chem Int Ed.

[105] Shu X-Z, Yang Y-F, Xia X-F, Ji K-G, Liu X-Y, Liang Y-M (2010). Platinum-catalyzed cross-dehydrogenative coupling reaction in the absence of oxidant. Org Biomol Chem.

[106] Xu C, Zhang L, Luo S (2015). Catalytic asymmetric oxidative α-C–H N, O ketalization of ketones by chiral primary amine. Org Lett.

[107] Sud A, Sureshkumar D, Klussmann M (2009). Oxidative coupling of amines and ketones by combined vanadium- and organocatalysis. Chem Commun.

[108] Xiao J (2012). Merging organocatalysis with transition metal catalysis: highly selective α-alkylation of aldehydes. Org Lett.

[109] Sun S, Mao Y, Lou H, Liu L (2015). Copper(II)/amine synergistically catalyzed enantioselective alkylation of cyclic *N*-acyl hemiaminals with aldehydes. Chem Commun.

[110] Li Y, Zhang R, Bi X, Fu J (2018). Multifunctionalization of unactivated cyclic ketones via synergistic catalysis of copper and diarylamine: access to cyclic α-enaminone. Org Lett.

[111] Huo H, Fu C, Wang C, Harms K, Meggers E (2014). Metal-templated enantioselective enamine/H-bonding dual activation catalysis. Chem Commun.

